# Continuous ES/Feeder Cell-Sorting Device Using Dielectrophoresis and Controlled Fluid Flow

**DOI:** 10.3390/mi11080734

**Published:** 2020-07-29

**Authors:** Yuuwa Takahashi, Shogo Miyata

**Affiliations:** 1 Graduate School of Science and Technology, Keio University, 3-14-1 Hiyoshi, Kohoku-ku, Yokohama 223-8522, Japan; yuuwa-takahashi@z6.keio.jp; 2 Department of Mechanical Engineering, Faculty of Science and Technology, Keio University, 3-14-1 Hiyoshi, Yokohama 223-8522, Japan

**Keywords:** cell sorting, dielectrophoresis, fluid-induced shear force, pluripotent stem cell, feeder cell

## Abstract

Pluripotent stem cells (PSCs) are considered as being an important cell source for regenerative medicine. The culture of PSCs usually requires a feeder cell layer or cell adhesive matrix coating such as Matrigel, laminin, and gelatin. Although a feeder-free culture using a matrix coating has been popular, the on-feeder culture is still an effective method for the fundamental study of regenerative medicine and stem cell biology. To culture PSCs on feeder cell layers, the elimination of feeder cells is required for biological or gene analysis and for cell passage. Therefore, a simple and cost-effective cell sorting technology is required. There are several commercialized cell-sorting methods, such as FACS or MACS. However, these methods require cell labeling by fluorescent dye or magnetic antibodies with complicated processes. To resolve these problems, we focused on dielectrophoresis (DEP) phenomena for cell separation because these do not require any fluorescent or magnetic dyes or antibodies. DEP imposes an electric force on living cells under a non-uniform AC electric field. The direction and magnitude of the DEP force depend on the electric property and size of the cell. Therefore, DEP is considered as a promising approach for sorting PSCs from feeder cells. In this study, we developed a simple continuous cell-sorting device using the DEP force and fluid-induced shear force. As a result, mouse embryonic stem cells (mESCs) were purified from a mixed-cell suspension containing mESCs and mouse embryonic fibroblasts (MEFs) using our DEP cell-sorting device.

## 1. Introduction

Regenerative medicine is a remarkable new approach that restores damaged tissues or organs in the human body by constructing three-dimensional tissues with cells and scaffold material. Pluripotent stem cells (PSCs) are considered as a promising cell source for regenerative medicine because of their high proliferation rate and pluripotency [1,2]. Although PSCs show the potential to differentiate multiple kinds of cells to regenerate biological tissues and organs, it is difficult to establish a PSC culture so as to maintain their pluripotency because it is easily lost by disturbing the cell passage procedure and changing the physical culture conditions (vibration, temperature, etc.). Usually, PSCs have been cultured on feeder cells [2], matrix-coated substrates [3,4,5], or surface-modified substrates [6,7,8] to maintain their pluripotency. Recently, cell-adhesive matrix coating has become a major approach to culture PSCs: gelatin or Matrigel for mouse embryonic stem cells (mESCs) and induced pluripotent stem cells (iPSCs), and Matrigel or laminin fragments (LN-511, LN-511-E8, etc.) for human iPSCs [9,10,11,12]. However, in the conventional method, the PSC culture is still effective on the feeder cell layer because this method is superior in terms of the proliferation rate and pluripotency stability of the culture. Nevertheless, in the PSC culture on feeder layers, the contamination of feeder cells is not negligible when the PSCs are corrected for clinical or experimental use, such as cell sampling (gene and protein assay, histological analysis, etc.) and cell passage. Therefore, a simple and rapid method to eliminate feeder cells from the PSC culture in the cell collection process is required. Moreover, it is preferable to perform this method under damage-less and label-free conditions.

A major method for cell sorting is fluorescence-activated cell sorting (FACS) [13,14]. For the FACS method, the cells are labeled with fluorescent dyes to distinguish the type and function of cells. The FACS system detects the fluorescence of labeled cells one-by-one; therefore, the sorting accuracy is high. Another method is magnetic-activated cell sorting (MACS) [15]. The MACS system distinguishes cells using a magnetically labeled antibody. These conventional methods are superior in terms of accuracy and efficacy; however, immunological or fluorescent staining is required to label the cells [16,17,18]. From this perspective, we focused on dielectrophoresis, which handles the movement of cells by electrical force only.

Dielectrophoresis (DEP) is one of the most promising approaches for manipulating and separating cells. DEP is a phenomenon that occurs under an applied non-uniform electric field, inducing dipoles within a polarized cell in a buffer solution. The cell in a non-uniform electric field can be manipulated by DEP forces to make it move toward high or low electric field regions, depending on the relative electric property of the cells, which is related to the cell type and function. Therefore, the cell type and function can be distinguished based only on electrical properties, without any fluorescent staining or magnetic antibodies.

Many studies reported cell sorting devices using DEP phenomena to distinguish and manipulate various cells (stem cell, blood cell, cancer cell, etc.) [19,20,21,22,23,24,25,26,27,28,29,30,31,32,33,34,35,36]. These studies manipulate or separate the cells based only on the difference in DEP properties of cells, without any fluorescent staining and magnetic antibodies like FACS or MACS systems. Single cell manipulation could be performed using dielectrophoresis [19,20]. As for the cell sorting technology, living cells and other particles could be separated using dielectrophoresis [21,22,23]. The DEP sorting of living cells according to cell properties (function, kind, size, etc.) has also been performed; however, these approaches required a three-dimensional electrodes array or microfluidic flow control with a numerical simulation and validation [24,25,26,27,28,29,30,31,32,33,34]. In addition, some researchers reported accurate cell sorting methods for blood cells or oocytes [35,36]. However, the continuity of the sorting process was not sufficient. In our previous study, a novel simplified cell manipulating and sorting device using DEP and fluid-induced shear force was developed [37,38,39]. It consisted of a transparent parallel-lined interdigitated-electrode array on an indium-tin-oxide (ITO)-coated slide glass. The accuracy and simplicity of our sorting device was sufficient; however, the continuity of the sorting was not. The aim of this study is to develop a simple and continuous cell sorting system to discriminate cells based on the difference in DEP properties using a liquid flow control system.

## 2. Materials and Methods

### 2.1. Dielectrophoresis

For a spherical particle in a non-uniform electric field, the time-averaged DEP force is generated on the particle as:

(1) $$F_{\mathrm{DEP}}=2\pi r^{3}\varepsilon_{0}\varepsilon_{m}Re\left[f_{cm}\left(\omega \right)\right]\nabla E_{rms}^{2}$$

where *r* is the radius of the microparticles, *ε*_0_ is the vacuum permittivity, *ε*_m_ is the relative permittivity of the surrounding medium, *Re*[*f*_cm_ (ω)] is the real part of the Clausius–Mossoti (CM) factor, and *E*_rms_ is the root mean square value of the imposed electric field [40]. The CM factor is related to the magnitude and direction of the DEP force. If the CM factor is positive, the DEP force caused on the particle is directed toward the region of high electric field intensity (positive-DEP). Conversely, if the CM factor is negative, the DEP force is directed toward the region of low electric field intensity (negative-DEP).

The CM factor is expressed as:

(2) $$f_{cm}\left(\omega \right)=\frac{\varepsilon_{p}^{*}-\varepsilon_{m}^{*}}{\varepsilon_{p}^{*}+2\varepsilon_{m}^{*}}$$

where $\varepsilon_{p}^{*}$ and $\varepsilon_{m}^{*}$ are the complex permittivities of the microparticles and the suspended medium, respectively. Each complex permittivity is defined as follows:

(3) $$\varepsilon_{}^{*}=\varepsilon_{0}\varepsilon -\frac{j\sigma }{\omega }$$

where *ε* is the relative permittivity of the particle or surrounding medium, *σ* is the electrical conductivity, and *ω* is the angular frequency of the applied AC electric field. This equation shows the dependency of the CM factor on not only the electric properties of the particle and surrounding medium but also on the frequency of the applied AC electric field. The frequency where the direction of the DEP force changes from n-DEP to p-DEP is called the crossover frequency. Our previous study reported that living cells and polystyrene beads could be separated based on DEP properties [19]. Therefore, cells could be distinguished based on differences in dielectrophoresis phenomena.

### 2.2. Cell Culture

In this study, mouse embryonic stem cells (mESCs) and mouse embryonic fibroblast (MEF) cells were used for the DEP cell sorting experiments. The mouse embryonic cell line, ES-B3, was obtained from Riken Bioresource center (Tsukuba, Japan), and the mitomycin C-treated MEF cells were from ReproCELL Inc. (Yokohama, Japan). The ES-B3 cells were cultured in 75-cm^2^ flasks in Glasgow Modified Essential Medium (GMEM) supplemented with 10% fetal bovine serum (FBS), antimycotics-antibiotics, and 1000 U/mL leukemia inhibitory factor (LIF). The MEFs were cultured in 75-cm^2^ flasks in GMEM supplemented with 10% FBS and antimycotics-antibiotics. Both cells were incubated in 5% CO_2_ and 95% humidity at 37 °C. Before the DEP experiments, the ES-B3 cells were passaged twice and MEFs were passaged once. Prior to the experiments, the cells were detached from the flasks using 0.05% trypsin and suspended in a low-conductivity buffer (LCB; 10 mM HEPES, 0.1 mM CaCl2, and 59 mM D-glucose in sucrose solution) [37,38,39]. The concentration of each cell suspension for DEP characterization was 5.0 × 10^6^ cells/mL, and the mixed ratio of ES-B3 and MEF cells for the DEP cell-sorting experiment was set at 4:6, according to a conventional on-feeder culture.

### 2.3. DEP Characterization of ES-B3 and MEF Cells

**Figure 1:**
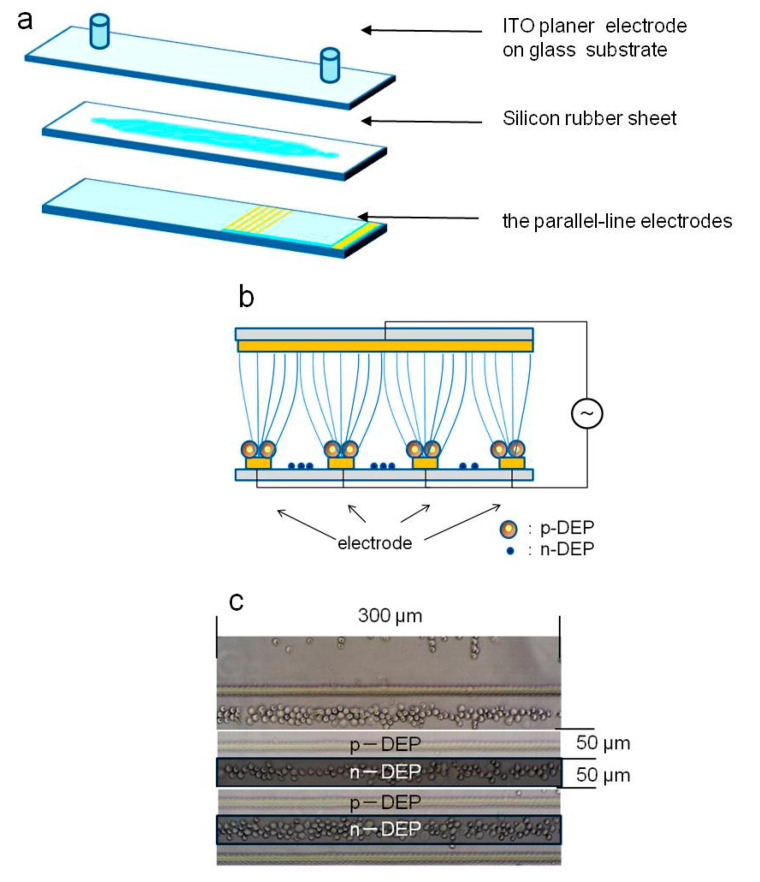
Dielectrophoretic characterization of living cells: (**a**) Schematic of dielectrophoresis (DEP) chamber; (**b**) Positive- and negative- DEP of living cells; (**c**) Discrimination of positive- and negative-DEP.

To sort ES-B3 and MEF cells from the mixed cell suspension, the DEP characteristics of ES-B3 and MEF cells were evaluated. To determine the crossover frequency between negative- and positive-DEP, the behavior of each cell was evaluated under various AC voltage frequencies. To cause the DEP phenomenon, a non-equal electric field was generated using transparent conductive glass (Figure 1) [37,39]. This chamber consisted of a transparent parallel-line electrode array on a glass substrate, ITO-coated glass, and a silicone rubber gasket. The parallel-line electrode array was fabricated using ITO-coated glass (Geomatec Co., Ltd., Yokohama, Japan) as a conductive substrate. The thickness of the ITO layer was 1500 Å, and the resistance was 5 Ω/sq. The parallel-line electrode was patterned using laser etching techniques. The electrode array was designed to generate a highly non-uniform electric field [37,39]. The width of each electrode line was 20 μm, and the spaces between each electrode were 80 μm (Figure 1a). The flow channel was made from a silicon rubber gasket to make a rectangular volume. The DEP chamber was formed by sandwiching the silicon rubber gasket between the parallel-line electrode array and a bare ITO-coated slide glass drilled with holes for the fluidic inlet and outlet. The thickness of the silicon rubber gasket was 500 μm. The cells were moved toward the electrodes by p-DEP and between electrodes by n-DEP in the DEP chamber (Figure 1b). The AC electric field was applied between the parallel-line electrode array and bare ITO-coated glass, using a function generator (WF1974, NF Corp., Yokohama, Japan) and amplifier (BA4850, NF Corp., Yokohama, Japan). The applied voltage was monitored by an oscilloscope (TDS1001B, Tektronix, Beaverton, OR, USA) connected in parallel. The movements of the cells within the DEP chamber were observed using a phase-contrast microscope (Nikon Eclipse TE300, Nikon, Tokyo, Japan) with a digital video camera.

For the DEP characterization, ES-B3 or MEF cell suspension in LCB was injected and subjected to an AC electric field 180 s after injection. The magnitude of the imposed AC voltage was 20 V_p-p_, and the frequency was varied from 10 kHz to 1 MHz. The behavior of ES-B3 and MEF cells was observed by the digital camera on the microscope, and microphotographs were captured 180 s after each AC voltage frequency was imposed. The captured images were trimmed to 200 μm × 300 μm, and the number of cells on the electrodes (positive-DEP) and between the electrodes (negative-DEP) were counted. The ratio of cells indicating positive-DEP in the chamber was calculated to evaluate the crossover frequency. The dielectrophoretic property of a cell (indicating p-DEP or n-DEP) was assessed based on the region where the cell moved (Figure 1c). The frequency dependency of the DEP property was also evaluated as:

Ratio of cells indicating positive-DEP = *N*_P_/(*N*_P_ + *N*_N_) (4)

where *N*_P_ and *N*_N_ are the number of cells indicating positive-DEP and negative-DEP, respectively. When the ratio of cells indicating positive-DEP is 50%, the frequency of the AC voltage is considered a transition point from negative-DEP to positive-DEP. In this study, the crossover frequency of the DEP is defined as the frequency at which the ratio of cells indicating positive-DEP is 50%.

### 2.4. Continuous Cell Sorting Using DEP and Fluid Shear Forces

**Figure 2:**
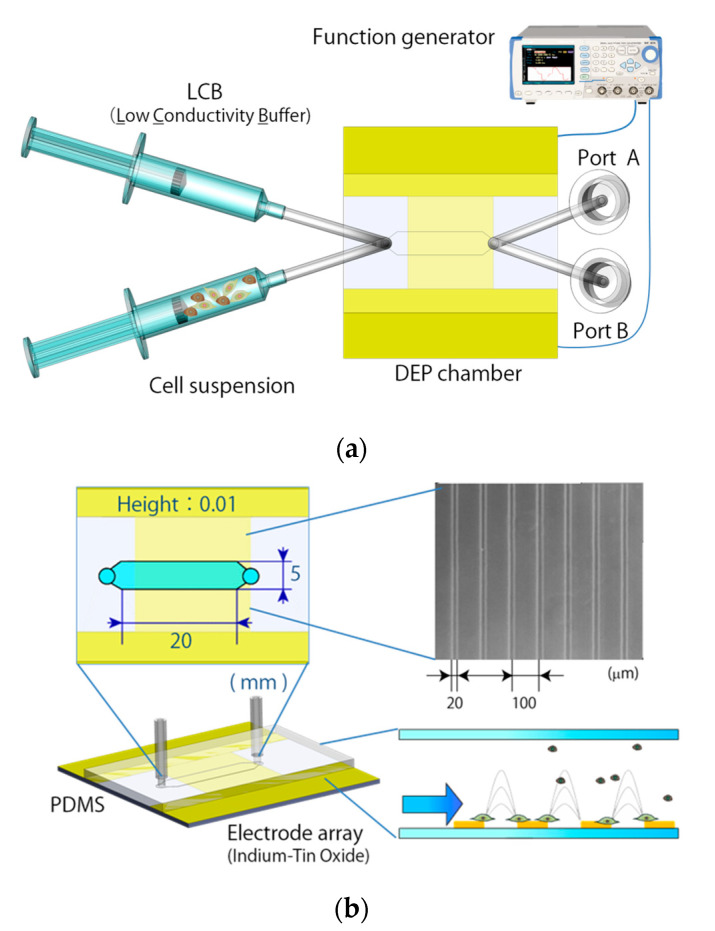
(**a**) Experimental setup for the DEP cell-sorting system combined with the fluid-control system; (**b**) Flow channel of the dielectrophoretic chamber and electrode array.

The DEP cell-sorting system consisted of a custom-made syringe pump with a PC control system, two syringes filled with the cell-mixed suspension containing ES-B3 and MEF cells and LCB solution, and the DEP chamber described in Section 2.3 (Figure 2a). The outlet of the DEP chamber was connected to two outlet ports, named port A (waste port) and port B (cell-collector port), through the switching channel. The DEP chamber was also connected to syringes containing cell suspension and LCB solution. The custom syringe pump controlled the flow rate of the cell suspension and LCB solution. The two syringes were connected to each other by a silicone rubber tube (diameter: 1 mm) and introduced into the DEP chamber. The DEP chamber for cell sorting had the same transparent parallel-lined electrode array on the lower surface of the chamber as described in Section 2.3. The flow channel of the DEP cell sorting chamber was made from a polydimethylsiloxane (PDMS) polymer sheet with a molded channel shape (Figure 2b). The DEP chamber was formed by a PDMS sheet sandwiched between the ITO glasses. The flow channel was designed to be 3 mm long from the inlet to the electrode array, 5 mm wide, and 100 μm high. The length of the flow channel was designed to ensure that whole cells were dropped on the electrode array during the cell-sorting procedure [20].

**Figure 3:**
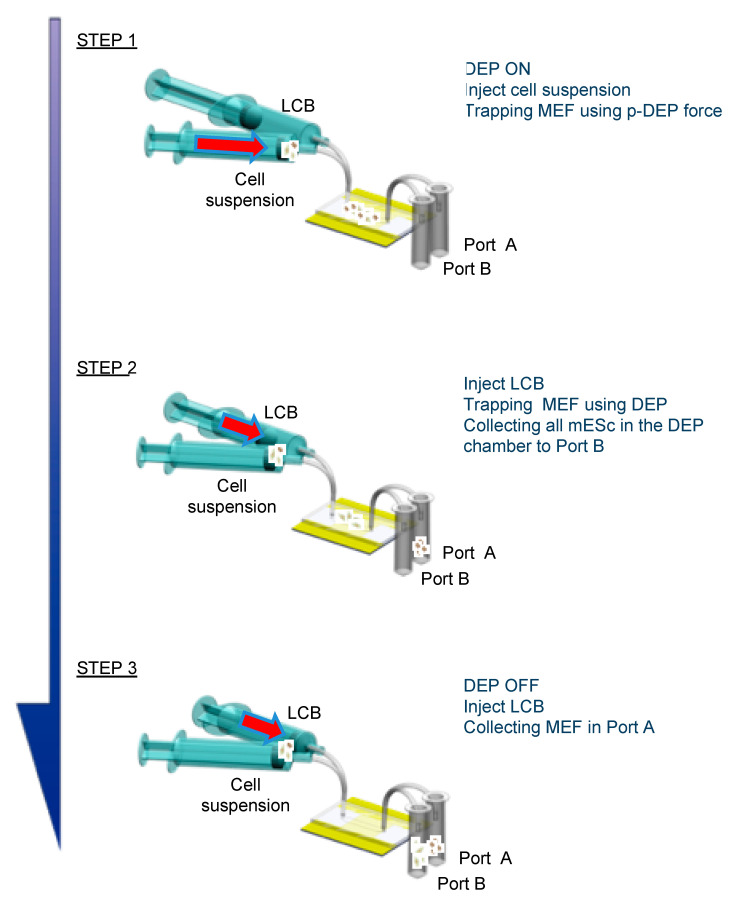
Sorting procedure using positive dielectrophoresis and fluid-flow control.

DEP and fluid-flow-based cell sorting was performed as described below (Figure 3). First, the cell-mixed suspension was injected into the chamber at a flow rate of 0.024 mL/min, and an AC electric field was imposed on the electrode array to cause p-DEP in MEF cells and n-DEP or neutral in ES-B3 cells (Step 1). In Step 1, the MEF cells were trapped on the electrodes, while the ES-B3 cells passed through the electrode array and were introduced into port B. After the line-electrode array was almost filled with the MEF cells, the AC voltage was switched off to remove the p-DEP force (Step 2). Next, the bulk LCB buffer was injected at a flow rate of 2.4 mL/min to release the trapped MEF cells from the electrode array, and they were introduced into port A (Step 3). To repeat this sorting procedure from steps 1 to 3, the MEF cells could be continuously eliminated from the ES-B3/MEF cell-mixed suspension.

Before the cell-sorting experiment, the DEP chamber was degassed and sterilized. To sterilize the chamber, the flow channel was filled with 70% ethanol for 5 min and washed twice with LCB solution. The DEP chamber was also filled with 0.2% bovine serum albumin (BSA) solution for anti-cell adhesion in the flow channel. Following the sterilization of the DEP chamber, the cell-mixed suspension was introduced into the chamber at a flow rate of 0.024 mL/min. In the sorting experiment, the AC electric field was set as 20 *V_p-p_* at 110 kHz to cause p-DEP for MEF cells only.

**Figure 4:**
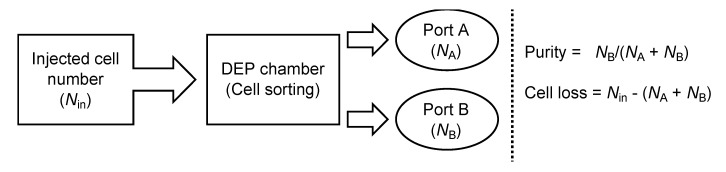
Evaluation of the purity of mouse ESCs and cell loss of sorted cells.

To evaluate the purification efficiency of the DEP cell-sorting system, the number of injected cells (*N*_in_) and those of ports A and B (*N*_A_, *N*_B_) were counted (Figure 4). The number of injected cells containing both ES-B3 and MEF cells was evaluated in order to compute the average number of cells passing through the first line of the electrode-array for 10 s. The numbers of cells in ports A and B were counted using a hemocytometer. The ES-B3 and MEF cells were distinguished based on the differences in the cell diameter; the diameter of ES-B3 cells ranged from 5–8 μm, and that of MEF cells ranged from 10–20 μm. The purification ratio of DEP cell sorting was determined so as to measure the existing ratio of ES-B3 cells in port B (collecting port). Moreover, the continuity of cell sorting was evaluated so as to measure the purification ratio at multiple cycles of our DEP cell-sorting system.

## 3. Results and Discussion

### 3.1. DEP Characterization of ES-B3 and MEF

**Figure 5:**
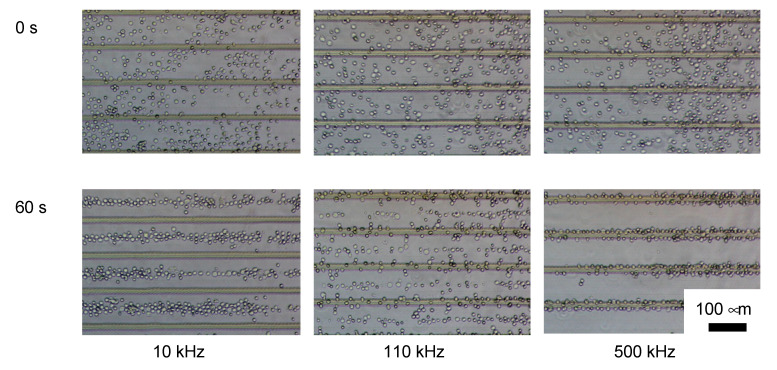
Dielectrophoresis of ES-B3 cells using a parallel line-electrode array before (0 s) and after (60 s) dielectrophoresis.

**Figure 6:**
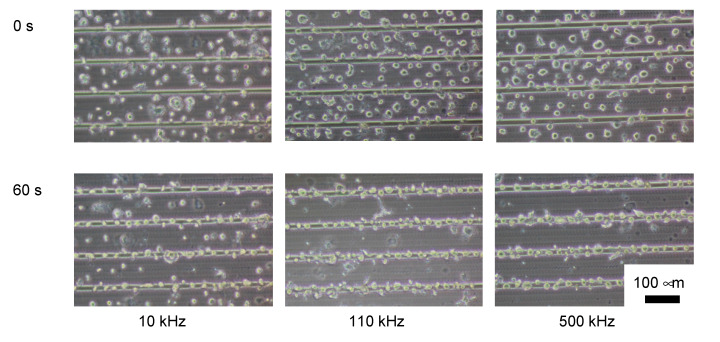
Dielectrophoresis of MEF cells using a parallel line-electrode array before (0 s) and after (60 s) dielectrophoresis.

**Figure 7:**
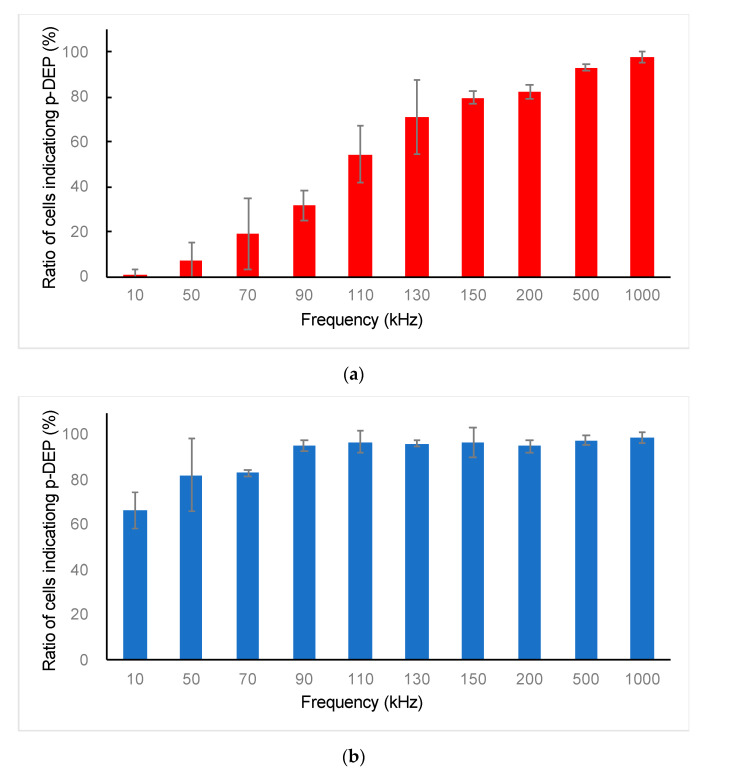
Ratio of (**a**) ES-B3 and (**b**) MEF cells indicating positive dielectrophoresis according to the frequencies of the applied AC electric field.

The ES-B3 cells showed n-DEP under 90 kHz of the AC electric field, whereas p-DEP was over 130 kHz (Figure 5). The MEF showed p-DEP continuously from 10 kHz to 1 MHz of the AC electric field (Figure 6). The ratios of ES-B3 and MEF cells indicating p-DEP are shown in Figure 7. The crossover frequency of ES-B3 cells was considered approximately 110 kHz, whereas that of MEF cells was under 10 kHz.

The dielectrophoretic property of living cells depends mainly on the electric property and diameter of the cells [40]. Therefore, it was considered that ES-B3 and MEF cells showed different dielectrophoretic properties because of their difference in cell size and functionality. Based on this difference in the DEP property, the ES-B3 and MEF cells could be distinguished under an AC voltage of 110 kHz, where the DEP force of the ES-B3 cell was negligible and the MEF cells showed a positive DEP and were trapped on the electrode array.

### 3.2. Purification Efficacy of DEP Cell-Sorting Device

**Figure 8:**
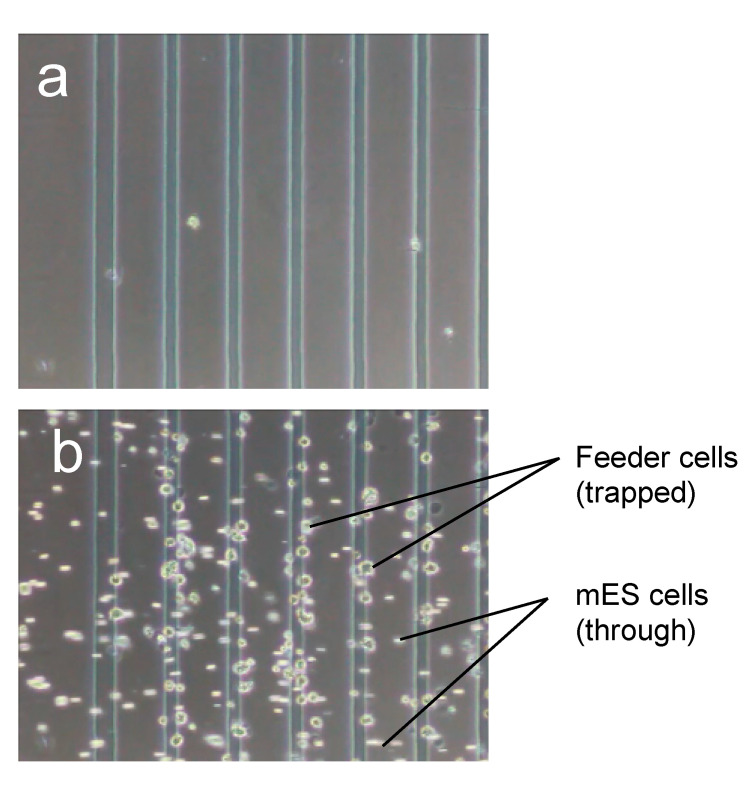

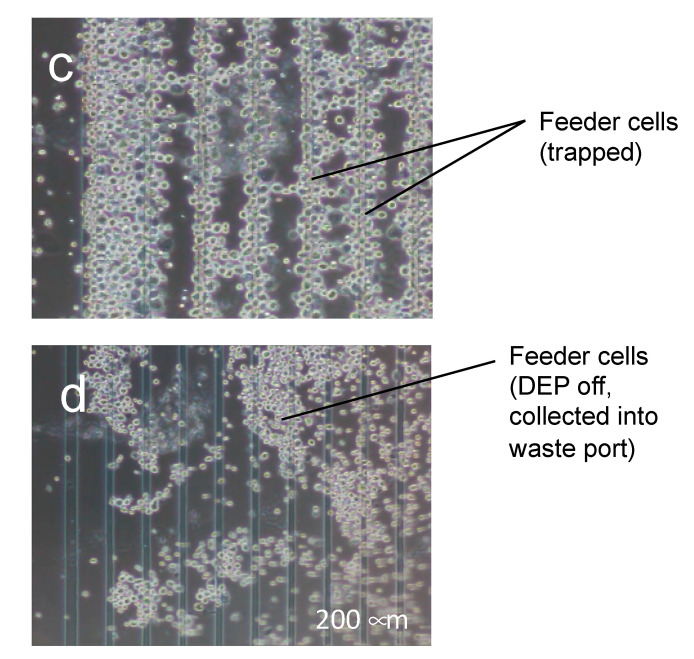
Purification of mESCs (ES-B3) from the ES/feeder cell-mixed suspension using DEP and fluid-flow control. (**a**) Injection of mixed-cell suspension; (**b**) Trapping of MEF cells by positive-DEP force and purification of ES-B3 cells from mixed-cell suspension; (**c**) Covering of 80% of the electrode array by MEF cells; (**d**) Release of trapped MEF cells.

**Figure 9:**
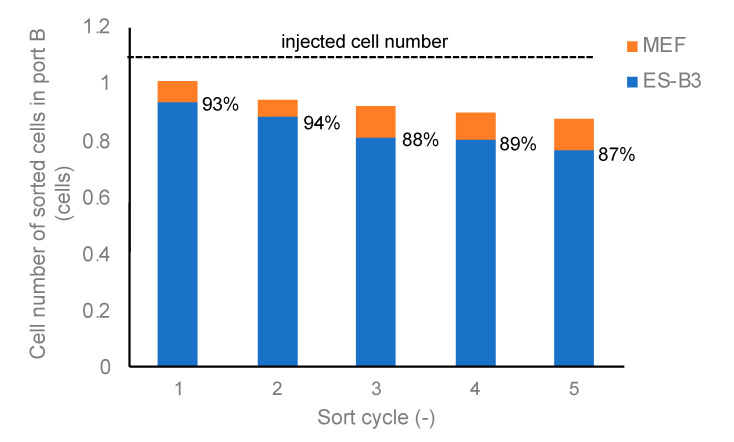
Purification ratio of ES-B3 cells in the sorted-cell suspension and total cell loss compared to the injected cell number.

In this study, a cell-sorting experiment on mESCs and feeder cells was performed using a DEP device combined with a fluid-flow control system, and the purification efficacy of mESCs was evaluated. During one cycle of cell sorting, the ES-B3 cells passed through the line-electrode array into port B; meanwhile, the MEF cells were trapped on the electrode array by p-DEP force and the trapped MEF cells were collected into port A (Video S1). Figure 8 shows the photomicrograph at each step of the DEP cell-sorting process. In step 1, the ES-B3/MEF-mixed cell suspension was injected into the DEP chamber (Figure 8a). Following step 1, the ES-B3 cells passed the line-electrode array, whereas the MEF cells were trapped on the electrode array by the p-DEP force against the fluid-induced shear force (step 2; Figure 8b). After 80% of the electrode array was covered by the trapped cells (Figure 8c), the p-DEP force was turned off in order to release and collect the MEF cells into port A (step 3; Figure 8d). Figure 8 shows the purification ratio of ES-B3 cells in port B for multiple cell-sorting cycles. The measured purification ratio of ES-B3 cells was about 59% before cell sorting and increased to about 94% in port B after the first cycle of cell sorting. The purification ratio decreased to approximately 90% during five cycles (Figure 9). The decrease in the purity of ES-B3 cells was derived from the residual cells in the corners of the chamber or the connective regions of the silicone rubber tubes. However, the purity of ES-B3 cells reached a plateau at approximately 90% and did not change after 10 cycles of cell sorting (data not shown). Therefore, the continuity and efficacy of DEP cell-sorting can be maintained if a large amount of cell sorting is required.

There are superior conventional cell-sorting methods, such as FACS and MACS, for evaluating the cell type or cell function quickly and accurately. Although these methods are effective for cell sorting, the labeling of cells by antibody or fluorescent dye is required [4,5]. Our newly developed DEP cell-sorting system does not require antibodies, fluorescence dyes, magnetic beads, or expensive equipment. In addition, the process of our DEP cell-sorting system was simple when compared to other DEP cell-sorting systems [41,42,43,44,45,46], with no complicated processes and with a high extensibility. Furthermore, our DEP cell-sorting system does not require a numerical analysis of the fluid-flow in the DEP chamber. By using a multiplex DEP-chamber or by extending the width of the chamber, the throughput of the DEP cell-sorting system could be larger. Therefore, our DEP-cell sorting system would be applicable for research and clinical uses requiring a large number of purified cells.

## 4. Conclusions

In this study, a novel DEP cell-sorting device using dielectrophoresis and fluid-induced shear force was developed in order to sort mouse embryonic cells and feeder cells. As a result, the feeder cells were eliminated from the ES/feeder mixed-cell suspension by DEP combined with fluid-induced shear force. Our device could also increase the purity of the mixed cell suspension in a continuous manner in order to process a large amount of cell suspension.

## Supplementary Materials

The following are available online at https://www.mdpi.com/2072-666X/11/8/734/s1, Video S1: Whole process of DEP cell sorting, from step 1 to step 3.
